# Association between competencies of biopsychosocial approach and job satisfaction of rehabilitation professionals in Ukraine

**DOI:** 10.1186/s12913-022-08755-z

**Published:** 2022-12-16

**Authors:** Aija Klavina, Kwok Ng, Olena Lazarieva, Marina Mruga, Kateryna Tymruk-Skoropad, Serhii Rokutov, Oleh Bazylchuk, Nataliia Zakaliak, Lorenzo Billiet, Lubov Tsizh, Dariya Popovych, Kateryna Myndziv, Olha Yezhova

**Affiliations:** 1 Latvian Academy of Sport Education, Brivibas Str. 333, Riga, Latvia; 2 European Federation of Adapted Physical Activity, Olomouc, Czech Republic; 3 National University of Physical Education and Science, Kyiv, Ukraine; 4 Lviv State University of Physical Culture, Lviv, Ukraine; 5 Pridneprovsk State Academy of Physical Culture and Sport, Kharkiv, Ukraine; 6 Khmelnytskyi National University, Khmelnytskyi, Ukraine; 7 Drohobych Ivan Franko State Pedagogical University, Lviv, Ukraine; 8 Katholieke Hogeschool VIVES, Kortrijk, Belgium; 9 Lviv State University of Physical Culture, Lviv, Ukraine; 10 I. Horbachevsky Ternopil National Medical University, Ternopil, Ukraine; 11 Horbachevsky Ternopil National Medical University, Ternopil, Ukraine; 12 Sumy State University, Sumy, Ukraine

**Keywords:** Biopsychosocial Approach, Job Satisfaction, Rehabilitation, Ukraine

## Abstract

**Background:**

The Biopsychosocial (BPS) model is recognized and widely accepted in the field of health care, particularly in rehabilitation. However, in clinical practice the applicability of the BPS model is a challenge for many professionals.

**Method:**

This study aimed to explore the factors that impact the perception of rehabilitation professionals about the BPS model in Ukraine. In addition, the job satisfaction assessment was done to measure whether rehabilitation specialists in Ukraine fulfil their professional roles according to their expectations and values. Participants were 346 rehabilitation specialists from Ukraine who completed the Bio-Psycho-Social Scale (BPS) for Use in Healthcare and the Global Job Satisfaction Scale (GJSS). The ANOVA was used to investigate the outcome differences between the two scales.

**Results:**

The highest proportion of responders in this study represented state health services. The BPS subscale outcomes on “assessment and reporting” and “professional knowledge and skills” were significantly higher for specialists from a private sector. The global job satisfaction scale outcomes did not differ between responders from the private and state health sector. The perception of professionals networks was associated with greater job satisfaction.

**Conclusions:**

The findings suggest that conception of the BPS approach in rehabilitation system of Ukraine varies across the private and state settings. The professional network plays important role in job satisfaction of rehabilitation professionals in Ukraine. Future research focusing on education strategies to effectively train professionals to apply biopsychosocial approach to practice is of critical importance to prepare rehabilitation specialists in Ukraine.

## Introduction

The International Classification of Functioning, Disability and Health (ICF) was launched by the World Health Organization [1], as a biopsychosocial model. It is a holistic approach that can assist health professionals on clinical reasoning, problem solving and goal setting. However, students and young professionals can find the ICF difficult to understand, particularly in how multiple biological, psychological, and social factors are associated with a persons’ health condition. Furthermore, in combination with other subjective factors, for example, their values, beliefs, cultural context, fears, worries, and hopes, the process of adopting the ICF can be challenging [2].

Linked with the use of the ICF, over the last decades there has been a shift towards a biopsychosocial (BPS) paradigm in health care and has resulted in the introduced concepts such as client-centered-practice, inclusion, shared decision making, coaching and self-management [3]. This has led to a switch from cure-oriented towards care-oriented practices. Yet, the implementation of the BPS model in rehabilitation has been slow, partly due to physiotherapists report lack of training and understanding of interventions according to the BPS in areas such as, musculoskeletal health problems [4], low back pain [5, 6], and traumatology [7]. Also, in occupational therapy, the introduction of the BPS model in rehabilitation has embraced a more integrated and holistic approach to practice [8, 9]. Moreover, the Rehabilitation Competency Framework (RCF), developed by theWHO expert Technical Working Group [10] provides a broad thematic organization for the competency, activity, knowledge and skill statements of the rehabilitation workforce across the five domains: (1) practice, (2) research, (3) management and leadership, (4) learning and development, and (5) professionalism. The RCF is intended to facilitate the development of context-specific competency frameworks, curriculum development, and competency-based workforce evaluation for rehabilitation [11].

Currently, Ukraine is one of the countries with high needs for rehabilitation services. The military conflict in the Donbass region since 2014 and the Russian large-scale invasion in Ukraine since February 24, 2022, has increased demands for acute and long-term rehabilitation services for many people injured in the war. According to the President of Ukraine, about 100 military personnel are killed every day and about 500 are wounded [12]. In addition, since 2015, there have been multiple structural and political changes in the rehabilitation system of Ukraine. For example, the physiotherapy profession did not exist until 2016. The Ministry of Health of Ukraine (MHU) established requirements for physiotherapists and physiotherapist assistants in [13, 14] and master study programs of physiotherapy and occupational therapy were separated by the MHU (2018). Since 2016, the implementation of ICF have been initiated by the MHU (Special Directive of Acting Minister of Health number 183, 2016; Directive of Cabinet of Ministers of Ukraine number, 2017). The ICF has been translated to Ukrainian, and Ukrainian legislation has been changed to enable implementation of the ICF across the country and ICF basic knowledge has been included in pre and post graduate study programs of healthcare professionals [15]. Also, train-the-trainers workshops for practitioners and policy making stakeholders were organized on ICF in 2018 (MHU, 2018), which were followed by implementation of the ICF in the Central eHealth database of Ukraine n 2020 [16]. However, multiple challenges related to national policy and provision of rehabilitation services limited implementation of the ICF. For example, a biomedical approach is still used in healthcare, professional recognition of physiotherapists and occupational therapists existed only since 2018, a lack of multidisciplinary rehabilitation services, and there is not available instruments to measure quality of rehabilitation services [17].

The noticeable factor of quality in health care systems is employee’s job satisfaction which further heavily influences the performance and clinical outcomes of patients. The studies exploring factors associated with job satisfaction in different groups of health care professionals have reported multiple components including job conditions, the work responsibilities, organisational policies, promotion opportunities, security, professional communication and relationship within the health care team [18]. Researchers have demonstrated the complex combination of organisational (work environment, culture, commitment, work demands and social support) and individual (burnout, demographic) factors on intention to leave the medical nursing profession [19, 20]. Furthermore, burnout was indicated as the most prevalent among medical professionals in the Middle East with highest rates for nurses reporting harsh work conditions, stress, and exposure to violence and conflict [21]. Information on job satisfaction among health care professionals including rehabilitation specialists in European countries is scarce. Eker et al. (2004) reported that leadership quality was one of the best predictors of job satisfaction among physiotherapists in Turkey. The other factors that showed strong relationship with job satisfaction were interpersonal relationships, income, opportunities for personal and professional growth, and professional advancement opportunities [22]. Other research reported that equipment and technologies available in the working environment, expectation to receive support from the organization, positive leadership style provide important insight into the type of work characteristic those can affect job satisfaction among health professional, including physiotherapists [23, 24].

Several projects funded by the European Commission have been initiated to promote collaboration with international rehabilitation partners aiming implementation of European standards of training for rehabilitation professionals in Ukraine. For example, the project entitled “Innovative Rehabilitation Education—Introduction of new master degree programs in Ukraine” (REHAB) (nr: 598,938-EPP-1–2018-1-LV-EPPKA2-CBHE-JP, http://rehabeukr.eu/) started in 2018 to address the three major goals related to development of innovative master programs in physiotherapy in Ukraine by: (1) building professional capacity of the academic and research staff, (2) development of the teaching/learning/assessment resources, and (3) providing specific educational infrastructure needed to implement the nationally new professional study program in physiotherapy. This project was committed to overcoming the traditional biomedical model, to promote biopsychosocial (BPS) approaches and to promote implementation of the ICF in Ukrainian rehabilitation services linked with the REHAB project. The academic staff and students from the four project partner universities in Ukraine (National University of Ukraine on Physical Education and Sport, Ivan Bobersky Lviv State University Of Physical Culture, Sumy State University and I.Horbachevsky Ternopil National Medical University) received more than 20 training sessions (webinars, seminars, onsite practical trainings and site visits) to learn a common language for describing physiotherapy as part of multidisciplinary rehabilitation system that involve different health professionals. The European project partners representing the Josef Pilsudski University of Physical Education in Warsaw (Poland) and European Federation of Adapted Physical Activity have more than 20 years educational and research experience in the rehabilitation field. More than one thousand bachelor and master students from physiotherapy programs and over one hundred academic and research staff from across Ukraine have been involved in the REHAB project training between 2018 – 2022.

Another project entitled Developing an Occupational Therapy study programme in Ukraine (nr. 609,589-EPP-1–2019-1-BE-EPPKA2-CBHE-JP), financed by Erasmus + Programme of the European Commission involves the three Ukraine higher education institutions (Drohobych Ivan Franko State Pedagogical University, Khmelnytskyi National University and Prydniprovsk State Academy) and European partners from Belgium (Vives University, Bruges) and Portugal (University of Porto). This particular project targets implementation of a new curriculum for occupational therapists according to the standards of the World Federation of Occupational Therapy (WFOT). In the project, the focus is not only on training the teachers but also the staff from hospitals and rehabilitation centers (Caritas and Modrychi), because they have a crucial role as guides and coaches of the internship students. Also, the collaboration with governmental institutions of Ukraine is promoted since their support is needed in state funded rehabilitation centers.

Although the use of ICF as a unified language and framework for a BPS description of health in various domains is supported by Ukrainian health policy, there is no data on the perception and understanding of the rehabilitation professionals in Ukraine on how they understand and if they use a BPS model in their clinical practice. Also, to the authors knowledge, no evaluation of job satisfaction of rehabilitation professionals has been carried out in Ukraine.

This study aimed to explore the factors that impact the perception of rehabilitation professionals about the BPS model in Ukraine. In addition, the job satisfaction assessment was done to measure whether rehabilitation specialists in Ukraine fulfill their professional roles according to their expectations and values.

## Methods

### Participants

This is a cross-sectional study of rehabilitation students and professionals in Ukraine. The data were collected through an online survey from a convenience sample of people who volunteered to take part in the study and met the inclusion criteria. The inclusion criteria for participation in the study included master level students in occupational therapy or physical therapy and people employed in rehabilitation. As there is currently no formal professional standard linked with the qualification needed for working in physical or occupational therapy in Ukraine, students were also recruited with working professionals. All respondents were from different regions of Ukraine (Lviv, Kyiv, Ternopil, Dnepropetrovsk, and Khelmitsky) and gave their consent to take part in the study. A total of 345 respondents were recruited, however 64 respondents had missed providing data on ‘years of experience’ and were removed from the analyses. After cleaning the data for missing values, there were 281 respondents (male = 31.8%, female = 68.2%). To preserve anonymity of the respondents (some were students from partner institutions), we did not track location data and do not know the spread of the distribution. The BPS scores of missing 64 responses (mean = 3.67, SD = 0.53) were tested against the remaining 281 responses (mean = 3.86, SD = 0.37) and after testing differences by independent t-tests, the differences were statistically significant (p = 0.010, d = 0.41), hence we conclude there is a potential sample bias with the participants who completed the survey.. This study complies with the Declaration of Helsinki and was performed according to ethics committee approval at the Latvian Academy of Sport Education, Ethics committee in Health Care (Nr.1, 2022.). Informed consent was obtained from all participants in this study. Data were collected between December 2021 – February 2022.

### Measures

The Bio-Psycho-Social Scale for Use in Healthcare [25] includes 31 questions based on five subscales. These five subscales with examples of the wording follows: (1) the competencies and the support in networking (BPS_N; 7 items, “I discussed the clinical decisions with my colleagues”), (2) the level of using expertise of the client (BPS_E; 7 items, “I used the lived experience in activities of daily living of the client in clinical decision making”. (3) the level of assessment and the coherent way of reporting (BPS_A; 4 items, “I used assessment tools to monitor the client wishes”), (4) the level of using professional knowledge and skills (BPS_P; 6 items, “I used my professional knowledge in clinical decision making”), and (5) the competence to use the environment in clinical decision making (BPS_U, 6 items, “We invited the client (and his family) to discuss the therapy”). All items had a five-point agreement Likert scale with 1 = strongly disagree, 5 = strongly agree. For each subscale, the mean values of the items were calculated for subscale analyses and the Cronbach alpha (α) was measured for the strength of the bio-psycho-social factors from the final sample. BPS_N (α = 0.631) had the lowest strength, and other factors had acceptable construct validity; BPS_E (α = 0.780), BPS_A (α = 0.739), BPS_ P (α = 0.830) and BPS_U (α = 0.731).

The Global Job Satisfaction Scale has nine-subscales that measure employee job satisfaction [26] in 36 items. Subscales include, pay, promotion, supervision, benefits, contingent rewards, operating procedures, co-workers, nature of work, and communication. Items were either positively or negatively worded with a six-point Likert agreement scale (1—disagree very much, 6—agree very much). Negatively worded items were reverse scored and an overall means score was created. As a global scale with all the items, the Cronbach alpha (α = 0.915) was high, therefore sub-scales were not used for further analyses.

Background variables included questions about the participant’s gender (male, female), qualifications (none, bachelors, masters, PhD or MD), work place setting (state health institution, private health institutions, private practice, or not applicable), as well as profession (rehabilitation medicine specialists, occupational or physiotherapists, or students). All items underwent a back-translation protocol whereby translators from English to Ukrainian did not share the original work to the translator who carried out the back translation. Where there were minor (e.g. cultural differences between ‘workload hours’ and ‘work schedule’) and major (e.g., the word treatment is different from therapy) discrepancies between original and translated text in the first round (n = 13), the researchers met to discuss the terms that appeared different in the translated text. After confirming with translators of the revised text, the questionnaire was uploaded to an online survey platform in both English and Ukrainian languages for completion. Data converted into English language and downloaded for analyses 28].

### Statistical analyses

Data analysed by IBM SPSS (version 27.0). Respondents were grouped by the SPSS two-step cluster command, where Bayesian Information Criterion (BIC) were used to find the best ratio between the BIC and the minimum number of clusters in the first step. The second step measures the distances between the clusters to produce the final set of clusters [27]. Workplace setting, profession, and qualification were entered into the algorithm, with AIC and Euclidean distances set to sort the clusters from the whole data set. The advantage of the two-step clustering is to examine the commonalities from within the data, and thus reduce the need for sampling weights or investigate representativeness of a convenience sample [28]. The clusters were then labeled for reporting purposes and it was agreed by the researchers on how to name them by examining the strongest predictor for the cluster as well as the characteristics from within the cluster.

To investigate the differences in BPA and global job satisfaction by clusters, mean scores of overall BPS and its components were tested by one way analysis of variance (ANOVA). Homogeneity of variances were tested with Levine’s test and because the null hypothesis was rejected (p = 0.915), the Tukey post-hoc test was carried out to control for Type 1 errors between the clusters (Field, 2017). In addition, parametric correlations of the BPA and global job satisfaction were performed to detect possible multicollinearity for linear regression analysis. To examine how BPA components were associated with global job satisfaction, individual linear regression analyses were performed for each cluster, where global job satisfaction was the dependent variable and each BPS component were the independent variables, after adjusting for background variables.

## Results

The four clusters were labeled as ‘None or Private Practice’, ‘Private health’, ‘OT or PT in Public Health’, and ‘Specialists in Public Health’. The characteristics of the clusters can be seen in detail from Table 1.

**Table 1:** Clusters of respondents and their characteristics

	Total %	None or Private Practice %	Private Health %	OT or PT in Public Health Institute %	Specialists in Public Health Institute %
	*N* = 281	*n* = 50	*n* = 72	*n* = 86	*n* = 73
Age mean (SD)	33.5 (11.9)	28.3 (9.5)	27.0 (7.2)	32.2 (10.6)	45.1 (10.4)
*Gender*					
Male	32.0	38.0	48.6	26.7	17.8
Female	68.0	62.0	51.4	73.3	82.2
*Work Setting*					
State health institution	51.6	2.0	0.0	100.0	79.5
Private Health Institution	28.5	10.0	100.0	0.0	4.1
Private practice	13.9	60.0	0.0	0.0	12.3
Not applicable	6.0	28.0	0.0	0.0	4.1
*Profession*					
Students	7.8	38.0	4.2	0.0	0.0
OT or PT	63.0	62.0	83.3	100.0	0.0
PRMS^a^	29.2	0.0	12.5	0.0	100.0
*Qualifications*					
None	3.6	10.0	6.9	0.0	0.0
Bachelors	29.9	48.0	34.7	27.9	15.1
Masters	42.3	36.0	52.8	62.8	12.3
PhD or medical doctor degree	24.2	6.0	5.6	9.3	72.6

^a^ PRMS physical and rehabilitation medicine specialists

### Main results

Across the four clusters, the mean scores for dimensions of BPS_A (Assessment and report) (F = 3.79, p = 0.011) varied across the clusters (Table 2). There were statistically significant post hoc differences between private health and specialists in public health (p = 0.005), but not across the different tests in any of the BPS factors or job satisfaction.

**Table 2:** Differences in mean scores (ANOVA) for BPS, each BPS dimension and global job satisfaction by cluster

	Total		None or Private Practice		Private Health		OT or PT in PHI		Specialist in PHI			
	Mean	SD	Mean	SD	Mean	SD	Mean	SD	Mean	SD	F	p
BPS	3.86	0.37	3.81	0.37	3.91	0.35	3.83	0.36	3.88	0.40	0.92	.432
BPS_P	3.99	0.46	3.90	0.51	4.01	.040	3.97	0.49	4.04	0.45	1.13	.337
BPS_A	3.88	0.57	3.91	0.40	4.03	0.50	3.86	0.61	3.72	0.66	3.79	.011*
BPS_E	3.96	0.49	3.89	0.51	4.07	0.49	3.92	0.45	3.92	0.51	1.92	.127
BPS_N	3.82	0.45	3.75	0.49	3.79	0.46	3.80	0.39	3.92	0.46	1.79	.148
BPS_U	3.59	0.57	3.56	0.58	3.61	0.52	3.55	0.60	3.64	0.59	0.38	.769
Job Satis	3.83	0.57	3.82	0.56	3.86	0.60	3.81	0.59	3.84	0.52	0.11	.957

BPS_P, Professional knowledge and skills; BPS_A, Assessment and report; BPS_E, Using expertise of the client; BPS_N, Networking; BPS_U; Using the environment; Job Satis, Global Job Satisfaction

*p < .05

Between the factors within BPS, the correlations ranged from 0.278 between BPS_P and BPS_N and 0.55 between BPS_U and BPS_N (Table 3).

**Table 3:** BPS component correlation Matrix

Correlation Matrix							
		BPS_PA	BPS_AA	BPS_EA	BPS_NA	BPS_UA	
BPS_PA	Pearson’s r	—					
	*p*-value	—					
BPS_AA	Pearson’s r	.501	—				
	*p*-value	< .001	—				
BPS_EA	Pearson’s r	.425	.536	—			
	*p*-value	< .001	< .001	—			
BPS_NA	Pearson’s r	.278	.309	.551	—		
	*p*-value	< .001	< .001	< .001	—		
BPS_UA	Pearson’s r	.429	.437	.532	.341	—	—
	*p*-value	< .001	< .001	< .001	< .001	—	—

Regression onto global job satisfaction by BPS components can be seen in Table 4. For the None or Private cluster, statistically significant predictors were increasing BPS_P, BPS_A, and BPS_N. There were no associations for job satisfaction among those in the private health cluster. Individuals in the OT or PT in Public Health Institutes had higher job satisfaction as BPS_N increased (p = 0.030). There were also a positive association between BPS_N (p = 0.006) and BPS_U (p < 0.001) and job satisfaction for specialists in public health institutes.

**Table 4:** BPS component standardised beta coefficients predicting global job satisfaction by cluster

	None or Private		Private Health		OT or PT in Public health		Specialists in Public health	
	B	p	B	p	B	p	B	p
BPS_P	.589	< .001*	.092	.527	.033	.783	-.107	.396
BPS_A	-.434	.005*	-.001	.996	.239	.783	.063	.637
BPS_E	.083	.598	.248	.167	.201	.065	-.038	.801
BPS_N	.443	.006*	.053	.693	.240	.107	.442	< .001*
BPS_U	-.017	.911	.081	.561	.024	.823	.397	.006*
F	6.65		2.33		7.36		9.38	
p		< .001		< .001		< .001		< .001
adj r^2^	.37		.09		.28		.37	

BPS_P, Professional knowledge and skills; BPS_A, Assessment and report; BPS_E, Using expertise of the client; BPS_N, Networking; BPS_U; Using the environment

*p < .05

## Discussion

### Key results

In this study, respondents to the survey were grouped into four clusters, mainly dominated by their workplace setting with about half of responders working in state funded health institutions. The National Health Service of Ukraine (NHSU) monitors and analyses the needs of citizens in health services and finances required for these services across the country. The NHSU implements the Program of Medical Guarantees since 2020 that covers 31 health benefit packages, including the three specifically related to rehabilitation: (1) medical rehabilitation for children aged 0–3 years, (2) persons with neurological conditions, and (3) persons with musculoskeletal conditions. For each rehabilitation package, the NHSU requires a rehabilitation staff to be available and issues the requirement for a multidisciplinary rehabilitation team, basic equipment, goal-setting principles and use of the ICF [17]. This explains the high proportion of responders in this study representing state health services. Regarding education level, the largest cluster were with master’s degree. It should be noted that master degree study programs in physiotherapy, were, for the first time in Ukraine,implemented during the REHAB project in 2019 at three universities of Ukraine in cities of Ternopol, Kiev and Lviv. Moreover, the only programme in occupational therapy was started in 2019 at the National University of Physical Education and Sport (NUPES), also a REHAB project partner. The growing number of graduates from rehabilitation related study programmes can also indicate trends in workforce numbers in future. However, there is a lack of detailed statistics on the number and location of rehabilitation specialists in Ukraine [17].

Regarding BPS scale results, the scores tend to be higher than in previous studies [3]. It might be explained by heterogeneity of the participants in other studies while this study involved only rehabilitation professionals. The Ministry of Health of Ukraine is focusing on raising the awareness on implementation of the ICF approach in education and in continuous professional development of rehabilitation staff in Ukraine [29]. However, the REHAB project has demonstrated that education of physiotherapists in Ukrainian is more focused on problem or disease oriented (International Classification of Disease, ICD) but not to the goal oriented approach (ICF).

More detailed analyses of the BPS survey outcomes across the four clusters indicated that significant differences were observed between the private and public health institutions for subscale “assessment and reporting” and “professional knowledge and skills” presenting significantly higher values for the rehabilitation specialists from a private sector. These results show that public health institutions do not have sufficient access to assessment instruments or do not use them accordingly. According to WHO reports about the situation of assessment of the rehabilitation system in Ukraine, it was stated that there are financial weaknesses related to limited rehabilitation packages in the NHSU monitored services that also includes availability of assessment instruments [17]. Also, the report indicated that private rehabilitation clinics are especially well equipped and therapists as well as patients were actively engaging in the therapy.

The global job satisfaction scale outcomes did not differ across the four clusters.

The current study showed that the rehabilitation specialists in Ukraine, whether working in the state or private sector, were uncertain with respect to satisfaction with their jobs.

The variables that contributed highly to the total scores of job satisfaction varied by cluster. The most notable predictor for job satisfaction was the BPS_N among the “None”, “OT or PT”, and “PRMS” clusters. In other words, increased perceptions of networks were associated with greater job satisfaction. Networks in physiotherapy can be good for improving cross-sectoral work, learning opportunities [30] and are reported to be associated withjob satisfaction [31]. The one dimension of the BPS that was not associated with job satisfaction was the BPA_E (using the expertise of the client). As clients have multiple experiences when visiting physiotherapists, it may be too broad of a phenomenon, and that is a reason for the lack of association to job satisfaction. Particularly, when communication with the client is often provided in training for physiotherapy [32], there might be mixed opinions across the four clusters.

The rehabilitation institutions in Ukraine have limited possibilities to meet the standards set by the NHSU for facilities and equipment. Also, there is limited access to continuous professional development (CPD) of in-service rehabilitation professionals. While CPD is required for medical doctors, it is not yet required for other professions within the rehabilitation workforce [17]. All above mentioned factors related to a work environment, support from employees and professional advancement are associated with job satisfaction of health specialists, including physiotherapists [22, 33]. Moreover, the lack of monitoring to measure job satisfaction of rehabilitation professionals has been indicated as one of the weaknesses of Ukrainian state (NHSU) services [17].

This study presented small to moderate correlations between BPS subscales. It might be linked with the perception of physiotherapists of learning and implementing the BPS approach that requires skills to comprehend the difference of this approach in order to applying their new skills. For example, [34] listed the five common themes of understanding the meaning of the biopsychosocial approach in the management of musculoskeletal conditions emerged from the patients’ and physiotherapists’ perceptions: (1) the difference of the new approach, (2) understanding pain, (3) patient-centered care, (4) gaining confidence, and (5) support.

The rehabilitation systems are different in every country and its conditions may depend on the cultural and socioeconomic environment in which specialists are trained, work and live. The implementation of the BPS approach for different group of patients requires team based and integrated service provisions with the holistic focus on clinical reasoning, problem solving and goal-setting [35]. In Ukraine the combination of two rehabilitation areas, physiotherapists and occupational therapists was used as one qualification until 2019. Furthermore, there is a need for highly qualified PT and OT professionals in universities to train new specialists. While there are 68 universities offering physiotherapy/ occupational study programs, each is different regarding content and quality of teaching. Moreover, speech and language therapists are not trained yet in Ukraine [17]. In summary, there is a need for transforming expertise across the Ukrainian rehabilitation system —first, in the rehabilitation system in general, and second, in relation to physiotherapists’ perception of this process of moving toward applying BPS oriented interventions in professional practice.

Regression analyses confirmed outcomes described above. For example, rehabilitation professionals reported higher job association with better access to assessment instruments, using professional network and possibility to use a variety of environment to contribute therapy plan (e.g., visiting and providing therapy for patient in his/her home environment). These outcomes are in line with previous studies reporting that working environment (including access to therapy assessment instruments) and relationship with patients are among important factors of job satisfaction of rehabilitation professionals [23, 22].

### Limitations

The study was cross-section in nature, therefore causality between variables cannot be established. The sampling was from a convenience sample from selected cities in Ukraine, therefore, interpretation of the results require caution prior to generalizing to all physiotherapists in Ukraine. While the study power can be considered sufficient, the number of participants was low and it is possible that a larger sample would have resulted in different proportion across the clusters, not least the differences in BPS scores between those who completed all questions and some (that were eventually excluded) may need to be considered when considering these results. Given the limitations and challenges described above, it seems that a deeper understanding is needed of rehabilitation professionals’ experiences of integrating BPS into clinical practice in Ukraine.

## Conclusions

The conception of the BPS approach in rehabilitation varies across the different settings and professionals affiliated with the Ukrainian health care system. In general, rehabilitation professionals, whether working in government or private hospitals, were positive about their job, while there were not fully satisfied scores. Overall, job satisfaction was associated with perception of professional networks. To gain further knowledge of the BPS implementation process across different rehabilitation settings in Ukraine, a qualitative study could be conducted in parallel with a feasibility study. Moreover, after the military aggression of Russia in Ukraine the health care challenges are affected by significant day to day increase of inpatient care related to war injuries in military personnel and civilians. The complex nature of military injuries requires unique expertise to develop new, innovative methods for trauma evaluation and treatment followed by decision of appropriate tactics of rehabilitation.

## Data Availability

The datasets used and/or analysed during the current study available from the corresponding author on reasonable request.
